# Targeted neuromodulation of pelvic floor nerves in aging and multiparous rabbits improves continence

**DOI:** 10.1038/s41598-021-90088-8

**Published:** 2021-05-19

**Authors:** Ana G. Hernandez-Reynoso, Dora L. Corona-Quintanilla, Kenia López-García, Ana A. Horbovetz, Francisco Castelán, Philippe Zimmern, Margarita Martínez-Gómez, Mario I. Romero-Ortega

**Affiliations:** 1 Department of Bioengineering, University of Texas at Dallas, Richardson, TX 75080 USA; 2 Department of Surgery, University of Texas Southwestern Medical Center, Dallas, 75390 USA; 3 Department of Biomedical Engineering and Biomedical Sciences, University of Houston, Houston, TX 77204 USA; 4 Centro Tlaxcala de Biología de la Conducta, Universidad Autónoma de Tlaxcala, Tlaxcala, Tlaxcala Mexico; 5 Departamento de Biología Celular y Fisiología, Unidad Foránea Tlaxcala, Instituto de Investigaciones Biomédicas, Universidad Autónoma de México, Tlaxcala, Tlaxcala Mexico; 6 Department of Urology, University of Texas Southwestern Medical Center, Dallas, 75390 USA; 7 Department of Health Care Sciences, University of Texas Southwestern Medical Center, Dallas, 75390 USA

**Keywords:** Biomedical engineering, Urology, Neural ageing, Peripheral nervous system

## Abstract

Pelvic floor muscle stretch injury during pregnancy and birth is associated with the incidence of stress urinary incontinence (SUI), a condition that affects 30–60% of the female population and is characterized by involuntary urine leakage during physical activity, further exacerbated by aging. Aging and multiparous rabbits suffer pelvic nerve and muscle damage, resulting in alterations in pelvic floor muscular contraction and low urethral pressure, resembling SUI. However, the extent of nerve injury is not fully understood. Here, we used electron microscopy analysis of pelvic and perineal nerves in multiparous rabbits to describe the extent of stretch nerve injury based on axon count, axon size, myelin-to-axon ratio, and elliptical ratio. Compared to young nulliparous controls, mid-age multiparous animals showed an increase in the density of unmyelinated axons and in myelin thickness in both nerves, albeit more significant in the bulbospongiosus nerve. This revealed a partial but sustained damage to these nerves, and the presence of some regenerated axons. Additionally, we tested whether electrical stimulation of the bulbospongiosus nerve would induce muscle contraction and urethral closure. Using a miniature wireless stimulator implanted on this perineal nerve in young nulliparous and middle age multiparous female rabbits, we confirmed that these partially damaged nerves can be acutely depolarized, either at low (2–5 Hz) or medium (10–20 Hz) frequencies, to induce a proportional increase in urethral pressure. Evaluation of micturition volume in the mid-age multiparous animals after perineal nerve stimulation, effectively reversed a baseline deficit, increasing it 2-fold (*p* = 0.02). These results support the notion that selective neuromodulation of pelvic floor muscles might serve as a potential treatment for SUI.

## Introduction

Stress urinary incontinence (SUI) is characterized by involuntary urine leakage during coughing, laughing or exercising, as elevation in abdominal pressure during those activities exceeds the urethral resistance, normally maintained by intrinsic and extrinsic sphincters^1,2^. This condition is often caused by injured or weakened pelvic floor muscles (PFM) which normally provide organ support and actively participate in urinary and fecal continence as secondary sphincters^3–6^. Damage to pelvic muscles often occur in pregnancy and childbirth, during which, soft tissue stretches approximately 147 ± 39%^7^, causing muscle injury as evidenced by changes in myofiber type composition, nuclei centralization, and fibrosis^8^. This type of injury to the pelvic floor muscles is exacerbated by aging^9,10^ and may include peripheral nerve injury, as the pudendal nerve has been estimated to stretch 13–35% during child delivery^11^, resulting in axon demyelination, partial muscle denervation, and impaired function^6,8,12^.

Our group has studied the role of pelvic floor muscles during micturition, parity and aging in adult female rabbits, and while no animal model fully mimics the human pelvic floor physiology, multiparous rabbits are a suitable model because of their well-developed pelvic floor musculature (i.e. in contrast with rats, rabbits have no vestigial pelvic floor muscles)^13–16^. In these animals, multiparity and aging result in PFM weakness, desynchronized muscle contraction patterns, and low urethral pressure and bladder efficacy^17–22^. Recently, we reported that nerve conduction in pelvic floor nerves is compromised in multiparous and aging rabbits, as evoked action potentials are reduced approximately 10–12% in young (12–18 months) and mid-age (3–4 years) multiparous animals compared to young nulliparous controls^18^. This indicated partial pelvic nerve damage in this animal model. However, the specificity and degree of nerve injury has not been fully investigated.

Here we use a comprehensive histomorphometric evaluation of pelvic and perineal nerves in the mid-age and multiparous rabbit model in order to define the degree of nerve injury. We hypothesized that multiparity and aging cause partial axon loss and demyelination, resulting in nerve excitability changes, including increase of the stimulation rheobase and chronaxie^23^, which may further contribute to pelvic floor muscles dysfunction and weakness.

In addition, we reasoned that acute electrical stimulation of partially injured pelvic nerves in the rabbit model would be able to modulate their function as an external urethral sphincter, serving as a potential treatment for SUI. Since electrical stimulation has been shown to activate the injured pudendal nerve in rats^24^, we evaluated the use of electrical stimulation of pelvic floor nerves, which branch from the pudendal nerve, to restore their urethral sphincter function. To that end, we used a miniature wireless cuff electrode for nerve stimulation, as reported previously^25,26^, and evaluated whether electrical stimulation of the pelvic nerves in mid-age multiparous rabbits, could evoke pelvic muscle contraction and partially reverse the reduced voiding efficiency and weak urethral pressure in this animal model.

## Results

### Pelvic nerve injury in multiparous and middle age animals

**Figure 1:**
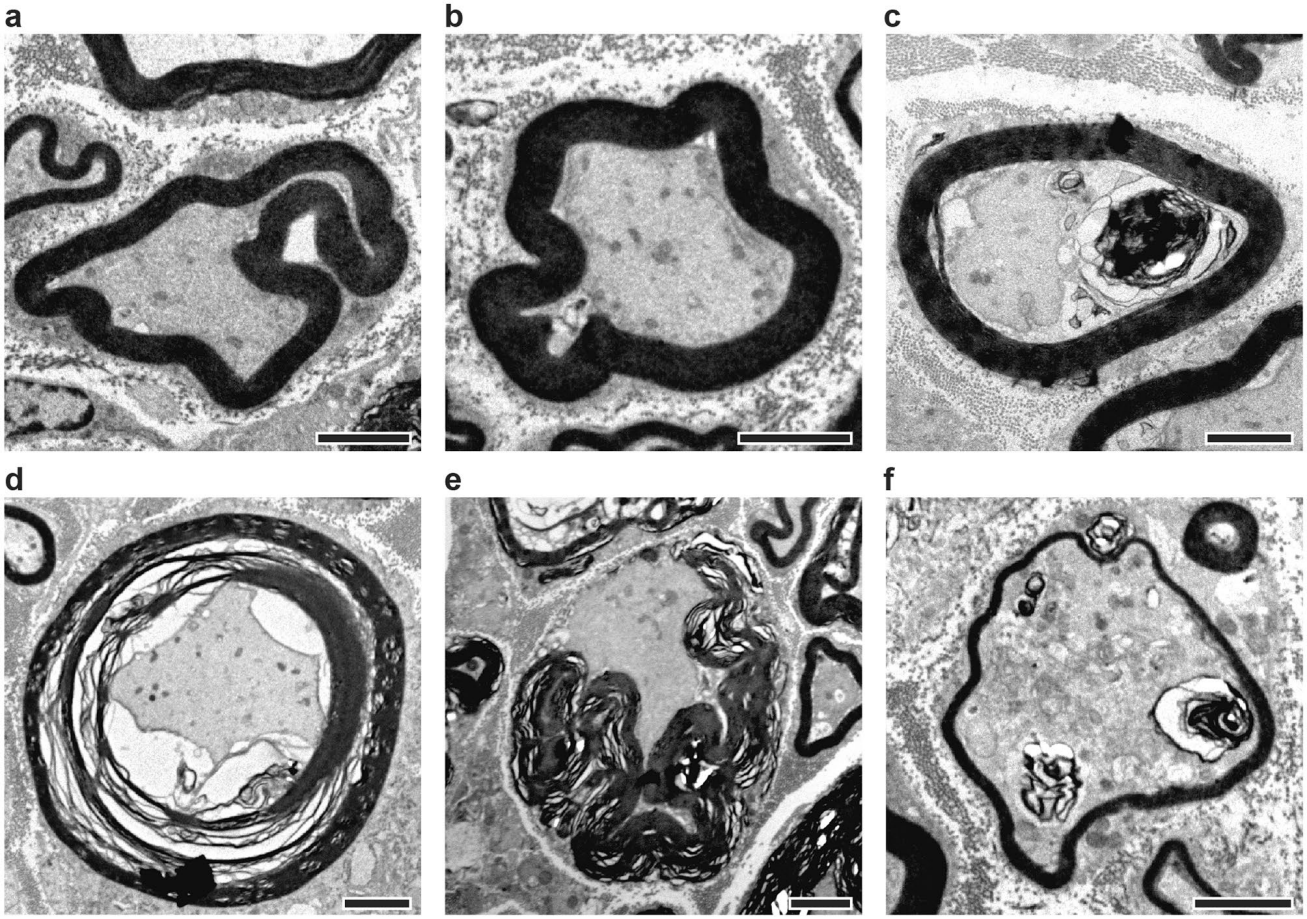
Representative TEM images of nerve injury and spontaneous regeneration in single axon fibers of the pelvic floor nerves. Myelin sheath abnormalities including: (**a**) infolding and (**b**) outfolding, (**c**) ovoid formation, (**d**) decompaction, and (**e**) disintegration. (**f**) Thin myelin on large myelinated axons, indicative of spontaneous regeneration. Scale bar = 2 µm.

The extent of injury was evaluated in a perineal nerve—the bulbospongiosus nerve—and a pelvic nerve—the pubococcygeus nerve—in young nulliparous, young multiparous, and middle-aged multiparous rabbits (in Table 1). The number of axons, circularity, and degree of myelination was analyzed using transmission electron microscopy (TEM). Qualitatively, normal axons in both nerves were circular with well-defined myelin sheaths. However, in multiparous and aging animals, we observed signs of nerve injury, including myelin looping with infoldings and outfoldings, ovoid formation (Fig. 1a–c), myelin decompaction (Fig. 1d), and axon disintegration indicative of Wallerian degeneration (Fig. 1e). Importantly, we also noticed the presence of axon fibers undergoing spontaneous regeneration, characterized by axon diameters larger than 6 μm with thin (i.e., < 0.5 μm), compacted myelin (Fig. 1f). These observations indicated a complex axonal composition with a mix of normal, injured and regenerated axon fibers in these nerves.

**Table 1:** Number of pregnancies, age, and weight per rabbit group.

	YN (n = 6)	YM (n = 7)	MM (n = 4)	*p*
Pregnancies	0	4	4	
Age (months)	11.3 ± 2.2	12.0 ± 5.3	43.1 ± 18.5***	0.0003
Weight (kg)	4.1 ± 0.6	3.7 ± 1.5	4.1 ± 0.4	0.75

All results are reported as mean ± SD; ***p < 0.001 as per Tukey’s post-hoc test for multiple comparisons (YM = young multiparous, MM = mid-age multiparous) versus young nulliparous (YN). The average litter size = 6.

### Axon histomorphometry of the bulbospongiosus nerve

#### Axonal composition

**Figure 2:**
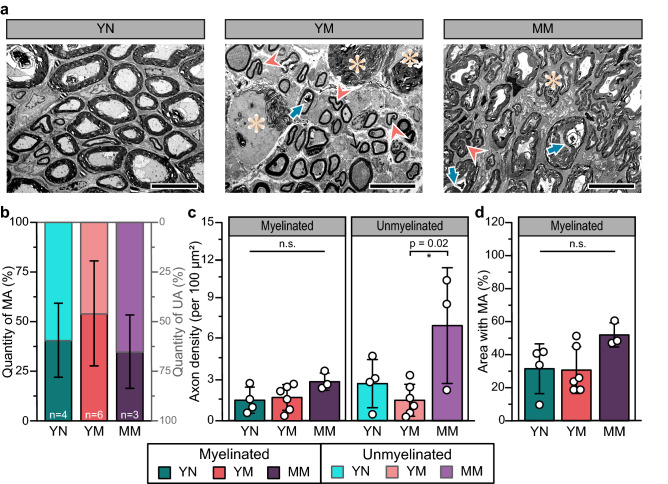
Axonal composition in the bulbospongiosus nerve. (**a**) Representative TEM images of cross-sections (Scale bars = 10 µm). Young multiparous (YM) and middle age multiparous (MM) rabbits showed signs of axon damage: thick arrow (blue) = myelin ovoid, arrowhead (orange) = recurrent loop, asterisk (beige) = Wallerian degeneration, and thin arrow (black) = unmyelinated axon bundle. (**b**) Quantity of myelinated (MA; left axis; dark colors) and unmyelinated (UA; right axis; light colors) axons as a percentage of total population shown as stacked bar plots; (**c**) Axon density per 100 μm^2^, and (**d**) Area of MA as a percentage of total area. Results are reported as mean ± SD, ns: no statistical significance and *p < 0.05 as per Tukey’s post-hoc test for multiple comparisons.

The bulbospongiosus nerve in young nulliparous animals consists mostly of large, myelinated axons. In contrast, the axons of this perineal nerve in multiparous animals were smaller in diameter and had clear myelination defects including myelin ovoids, recurrent myelin looping, and Wallerian degeneration (Fig. 2a). Table 2 shows the summary of the quantitative results from two investigators blinded to the rabbit groups on fiber diameter (*D*_*axon*_), myelin thickness, degree of myelination (G-ratio), and myelin morphology using the elliptical ratio (Φ) from electron microscopy cross-sectional images. The log-transformed data had a normal and homogeneous distribution, except for *D*_*axon*_ of the mid-age multiparous rabbits, although distribution density analysis showed that such deviation was not significant (Fig. 1 Supplementary). The percentage of bulbospongiosus myelinated axons in young nulliparous rabbits (40.5 ± 19%; Fig. 2b) was comparable to that of multiparous animals. Axon density in young nulliparous rabbits (1.5 ± 0.9 axons per 100 μm^2^; Fig. 2c) approximated 31.5 ± 15% of the total cross-sectional area (Fig. 2d) and was similar to that of young multiparous rabbits (1.7 ± 0.9 axons per 100 μm^2^). Conversely, we observed a mild increase in myelinated axons in the mid-age multiparous animals, estimating a 51.7% axonal density per cross section area. The number of unmyelinated axons in young multiparous animals (2.7 ± 1.8 axons per 100 μm^2^) were similar to that in young multiparous rabbits (1.5 ± 1.2 axons per 100 μm^2^.), but significantly increased in mid-age multiparous animals (Fig. 2c; 6.9 ± 4.2 axons per 100 μm^2^; *p* = 0.02), which may indicate demyelinated or regenerated axons.

**Table 2:** Summary of histomorphometry results for the bulbospongiosus nerve.

Bsn	p*	*D*_*axon*_	YN	YM	MM	*p*^$^	*p*^#^
Percentage of MA (%)	0.45	All	40.5 ± 19	53.9 ± 26	34.7 ± 19		
Percentage of UA (%)	0.46	All	59.5 ± 19	46.1 ± 26	65.3 ± 19		
MA density (per 100 μm^2^)	0.13	All	1.5 ± 0.9	1.7 ± 0.9	2.9 ± 0.6		
UA density (per 100 μm^2^)	0.02*	All	2.7 ± 1.8	1.5 ± 1.2	6.9 ± 4.2	0.69	0.02^**#**^
MA density coverage (%)	0.11	All	31.5 ± 15	30.7 ± 14	52.0 ± 7		
MA diameter (μm)	0.57	All	5.1 ± 2.0	5.0 ± 2.3	4.6 ± 1.7		
φ	0.46	≤ 5 μm	1.14 ± .08	1.13 ± .05	1.13 ± .03		
		> 5 μm	1.06 ± .02	1.07 ± .05	1.07 ± .02		
Thickness ( μm)	0.2E-3*	≤ 5 μm	1.9 ± 0.5	1.6 ± 0.3	2.7 ± 0.5	0.67	0.7E-2^**#**^
		> 5 μm	2.8 ± 1.0	2.9 ± 1.1	4.2 ± 0.6	0.99	0.19
G-ratio	0.2E-5*	≤ 5 μm	0.65 ± .02	0.65 ± .05	0.54 ± .06	0.84	0.4E-2^**#**^
		> 5 μm	0.72 ± .05	0.72 ± .06	0.61 ± .04	0.93	0.2E-2^**#**^

All results are reported as mean ± SD. p* = significance by MANOVA test. *p*^**$**^ = significance by Tukey’s post-hoc test between young multiparous (YM) and young nulliparous (YN). *p*^**#**^ = significance between mid-age multiparous (MM) versus YM. MA = myelinated axons, UA = unmyelinated axons.

#### Axon diameter and myelin looping

**Figure 3:**
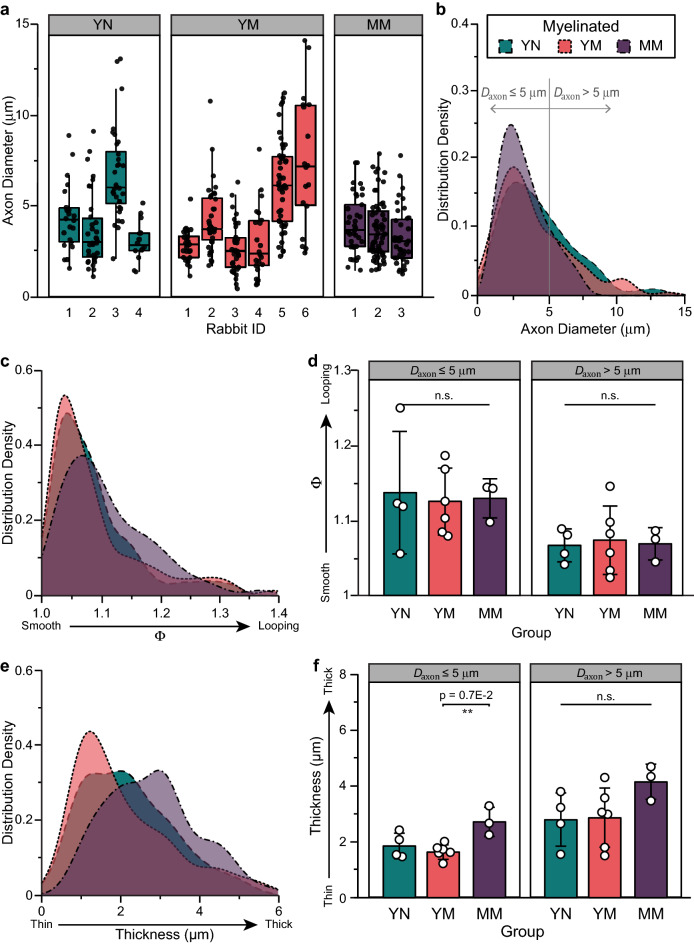
Histomorphometry analysis of the bulbospongiosus nerve. (**a**) Individual distribution of different axon diameter (*D*_*axon*_) in young nulliparous (YN), multiparous (YM) and mid-age multiparous (MM) rabbits, and its (**b**) distribution density per group. (**c**) Elliptical ratio (Φ) distribution (myelin looping) (**d**) its *D*_*axon*_-dependent average. (**e**) Myelin thickness distribution density and its (**f**) *D*_*axon*_-dependent average. Results are reported as mean ± SD; n.s: no statistical significance, **p < 0.007 as per Tukey’s post-hoc test for multiple comparisons.

The average diameter of bulbospongiosus myelinated axons (*D*_*axon*_) in young nulliparous rabbits was 5.1 ± 2.0 μm (Table 2 and Fig. 3a) and was comparable to that in multiparous rabbits (Fig. 3a; *p* = 0.57). However, axons larger than 7 μm in diameter were absent in the mid-age multiparous group (Fig. 3b). Myelin was mostly compacted in the nulliparous rabbits, with mild looping denoted by a trend towards increase in elliptical ratio (Fig. 3c), which was prevalent in the smaller axons (*D*_*axon*_ ≤ 5 μm; 1.14 ± 0.08), but not statistically different (Fig. 3d). In these control animals, the myelin thickness distribution (Fig. 3e) was skewed towards 2 μm and proportional to axon diameter (Fig. 3f). Axons with *D*_*axon*_ ≤ 5 μm had thinner myelin (1.9 ± 0.5 μm) compared to larger ones (2.8 ± 1.0 μm). In contrast, myelin thickness was found to be significantly increased in small axons (*D*_*axon*_ ≤ 5 μm) of mid-age multiparous rabbits (*p* = 0.007), consistent with the observed myelin decompaction in these animals. Together with the observed increase in the number of small unmyelinated axons (≤ 5 μm) in these animals (Fig. 2c), this indicates that approximately 12% of the axons are damaged in the bulbospongiosus nerve, which is consistent with the reduction in electrical activation thresholds previously reported^18^. The increase in myelination thickness on small diameter axons may be indicative of axonal regeneration.

**Figure 4:**
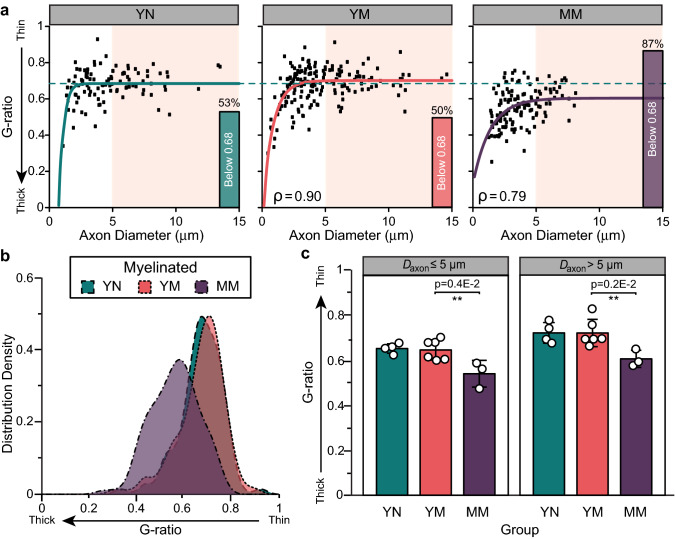
Bulbospongiosus nerve injury in mid-age multiparous (MM) animals evidenced by a decrease in G-ratio. (**a**) Scatter plot of G-ratio versus axon diameter (*D*_*axon*_) with non-linear regression [G-ratio_*group*_(*D*_*axon*_)]. The plateau (${\text{G-ratio}}_{plateau}^{group}$) of the G-ratio_*YN*_(*D*_*axon*_) is overlaid on the young multiparous (YM) and MM plots. Bars represent the percentage of axons that are below ${\text{G-ratio}}_{plateau}^{YN}$= 0.68. The correlation coefficient (ρ) between G-ratio_*YN*_(*D*_*axon*_) and the YM and MM scatter data is indicated at the bottom of each scatter plot. *D*_*axon*_ > 5 μm. (shaded area). (**b**) Distribution density histogram of G-ratio showed a decrease in the MM rabbits. (**c**) Averaged G-ratio in small (*D*_*axon*_ ≤ 5 μm) and large (*D*_*axon*_ > 5 μm) axons. All results are reported as mean ± SD; **p < 0.004 as per Tukey’s post-hoc test for multiple comparisons.

Evaluation of the myelinated axon diameter over that including the thickness of the myelin sheet is known as the g-ratio, and is interpreted as a reliable indicator of axon health. Values close to 0.6 are considered optimal for nerve conduction and those less than 0.4 indicate presence of damaged axons. In both nulliparous and multiparous young animals, the average G-ratio was approximately 0.68 with 50–53% of axons showing smaller values, indicating that multiparity did not drastically affected the axon myelination (Fig. 4a). However, mid-age multiparous animals showed a significant shift towards smaller g-ratios, where 87% of the axons showed reduced values, indicating axon injury (Fig. 4b). This decrement was significant in both small (*D*_*axon*_ ≤ 5 μm; *p* < 0.004) and large (*D*_*axon*_ > 5 μm; *p* < 0.002) myelinated axons (Fig. 4c).

### Histomorphometry of the pubococcygeus nerve

**Figure 5:**
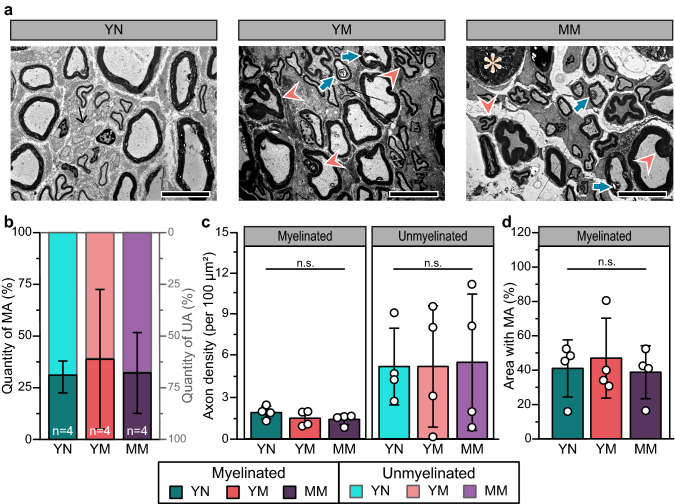
Analysis of axons in the pubococcygeus nerve. (**a**) Representative TEM images in cross-sections (Scale bar = 10 µm) for all rabbit groups. Changes in myelin morphology suggesting axon damage: thick arrow (blue) = myelin ovoid, arrowhead (orange) = recurrent loop, asterisk (beige) = Wallerian degeneration, and thin arrow (black) = unmyelinated axon bundle. (**b**) Quantification of myelinated (MA; left axis; dark colors) and unmyelinated (UA; rigth axis; light colors) axons as a percentage of total number of axons. (**c**) Axon density per 100 μm^2^ of both, myelinated and unmyelinated axons show consistent density among all rabbit groups. (**d**) Area coverage of myelinated axons as a percentage of total area. All results are reported as mean ± SD; n.s: no statistical significance, Tukey’s post-hoc multiple comparisons.

**Figure 6:**
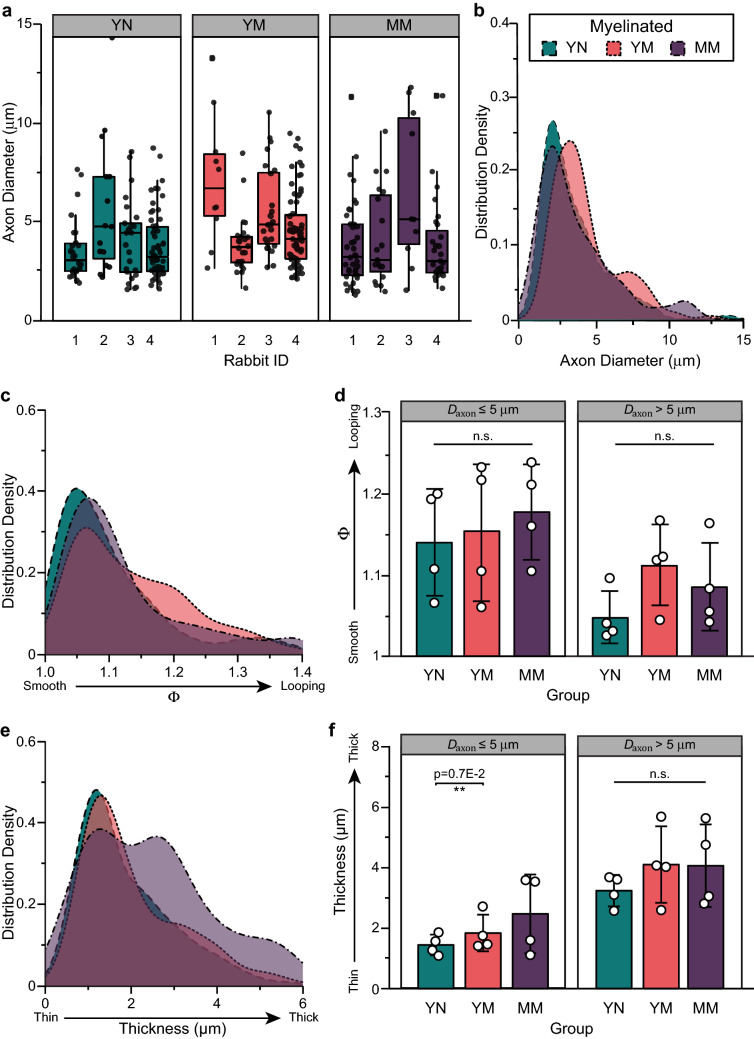
Analysis of the pubococcygeus nerve injury in rabbits indicating the (**a**) axon diameter (*D*_*axon*_) for all animals and its (**b**) distribution density. (**c**) Distribution density of elliptical ratio (Φ) and (**d**) quantification of its *D*_*axon*_-dependent average. And finally, (**e**) distribution density histogram of myelin thickness (*D*_*myelin*_—*D*_*axon*_) for all groups and its (**f**) *D*_*axon*_-dependent average. All results are reported as mean ± SD; n.s: no statistical significance, **p < 0.01, Tukey’s post-hoc multiple comparisons.

The pubococcygeus nerve in healthy, young nulliparous controls was mainly composed of large myelinated axons with no major gross abnormalities resulting from multiparity (Fig. 5a). Unlike the bulbospongiosus nerve, unmyelinated axons accounted for 69.6 ± 8% of the cross-sectional area (4.9 ± 2.6 per 100 μm^2^), while myelinated axons composed 40.7 ± 17% of the area at a density of 2.0 ± 0.5 6 per 100 μm^2^ (Fig. 5b–d). Pubococcygeus axons averaged 5.6 ± 2.3 μm in diameter in the young nulliparous rabbits (Fig. 6a), and those in mid-age multiparous and young multiparous rabbits were comparable. The density distributions of the myelinated axons in young nulliparous and mid-age multiparous overlapped, whereas those in young multiparous rabbits were skewed 0.8 μm towards larger axon diameters. We found that myelin looping was not prevalent in young nulliparous rabbits (Fig. 6c) averaging 1.14 ± 0.07 for *D*_*axon*_ ≤ 5 μm and 1.05 ± 0.03 for those with *D*_*axon*_ > 5 μm (Fig. 6d). These results indicated that the smaller axons in the young nulliparous animals were more irregular in shape and surrounded by thinner myelin (Fig. 6e,f). In turn, myelin looping was more prevalent in mid-age multiparous, mainly affecting the larger axons, albeit not significantly (*p* = 0.34). In the mid-age multiparous rabbits, myelin thickness was comparable in the larger axons (*p* = 0.20), but significantly thicker (*p* < 0.01) in smaller axons, suggestive of axonal regeneration.

**Figure 7:**
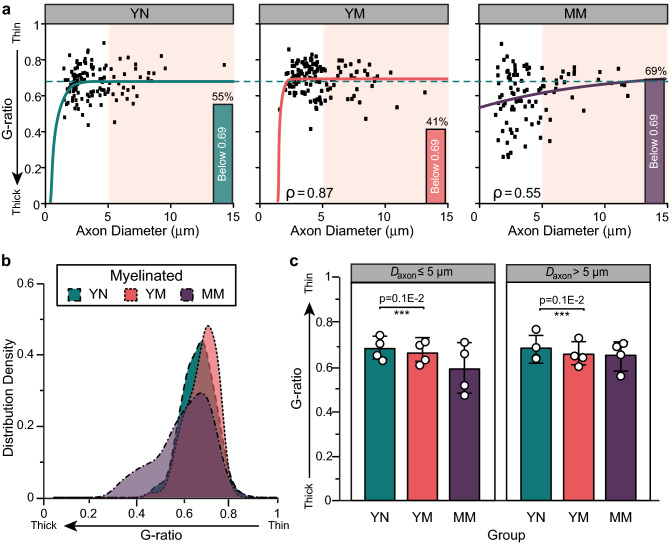
G-ratio distribution in the pubococcygeus nerve. (**a**) Scatter plot of G-ratio versus axon diameter (*D*_*axon*_) with non-linear regression [G-ratio_*group*_(*D*_*axon*_)]. The plateau (${\text{G-ratio}}_{plateau}^{group}$) of the G-ratio_*YN*_(*D*_*axon*_) is overlaid for comparison. Bars represent the percentage of axons that are below ${\text{G-ratio}}_{plateau}^{YN}$= 0.69. The correlation coefficient (ρ) between G-ratio_*YN*_(*D*_*axon*_) and the scatter data is indicated at the bottom of each scatter plot. *D*_*axon*_ > 5 μm (shaded orange region). (**b**) G-ratio distribution density histogram of young multiparous (YM) and mid-age multiparous (MM) overlaid with young nulliparous (YN) rabbits for comparison. (**c**) Quantification of the G-ratio stratified by *D*_*axon*_ (≤ 5 μm and > 5 μm). All results are reported as mean ± SD; ***p < 0.001, Tukey’s post-hoc test multiple comparisons.

As expected, the distribution of axon diameter and G-ratio in young multiparous rabbits was strongly correlated (ρ = 0.87; Table 2 Supplementary) to the young multiparous distribution. In contrast, mid-age multiparous animals showed a weak correlation (ρ = 0.55) with a significant dispersion of small axons with low G-ratios, indicating either myelin decompaction as a result of injury or axonal regeneration (Fig. 7a,b). Log-transformation analysis showed that these changes were statistically significant in the mid-age multiparous, compared the young animals (*p* < 0.001) in both small and large axons groups (Fig. 7c).

Together, these results revealed partial damage of the pelvic and perineal nerves in the rabbit model, primarily in the mid-age and multiparous animals, resulting in decompacted myelin and Wallerian degeneration of small and large axonal populations in these nerves. Signs of nerve regeneration were also observed, as indicated by large axons with thin myelin (Table 3).

**Table 3:** Summary of histomorphometry results for the pubococcygeus nerve.

Pcn	p*	Axon size	YN	YM	MM	*p*^$^	*p*^#^
Percentage of MA (%)	0.86	All	30.4 ± 20	40.0 ± 34	32.4 ± 20		
Percentage of UA (%)	0.86	All	69.6 ± 8	61.0 ± 34	67.6 ± 20		
MA density (per 100 μm m^2^)	0.34	All	2.0 ± 0.5	1.5 ± 0.6	1.4 ± 0.4		
UA density (per 100 μm m^2^)	0.86	All	4.9 ± 2.6	4.9 ± 4.1	5.2 ± 4.6		
MA density coverage (%)	0.82	All	40.7 ± 17	46.7 ± 23	38.5 ± 15		
MA diameter ( μm)	0.38	All	5.2 ± 2.3	5.6 ± 2.3	5.2 ± 2.6		
φ	0.34	≤ 5 μm	1.14 ± .07	1.15 ± .09	1.18 ± .06		
		> 5 μm	1.05 ± .03	1.11 ± .05	1.09 ± .05		
Thickness ( μm)	0.01*	≤ 5 μm	1.5 ± 0.3	1.8 ± 0.6	2.5 ± 1.3	0.7E−2^$^	0.53
		> 5 μm	3.2 ± 0.5	4.1 ± 1.3	4.1 ± 1.4	0.20	0.77
G-ratio	0.5E-10*	≤ 5 μm	0.69 ± .06	0.67 ± .05	0.60 ± .12	0.4E−4^$^	0.88
		> 5 μm	0.69 ± .06	0.66 ± .05	0.66 ± .07	0.3E−4^$^	0.75

Comparison as following: * = MANOVA, and Tukey’s post-hoc test for multiple comparisons, where ^**$**^ = young multiparous (YM) versus young nulliparous (YN), and ^**#**^ = mid-age multiparous (MM) versus YM. All results are reported as mean ± SD.

### Acute bulbospongiosus neuromodulation in mid-age multiparous animals

**Figure 8:**
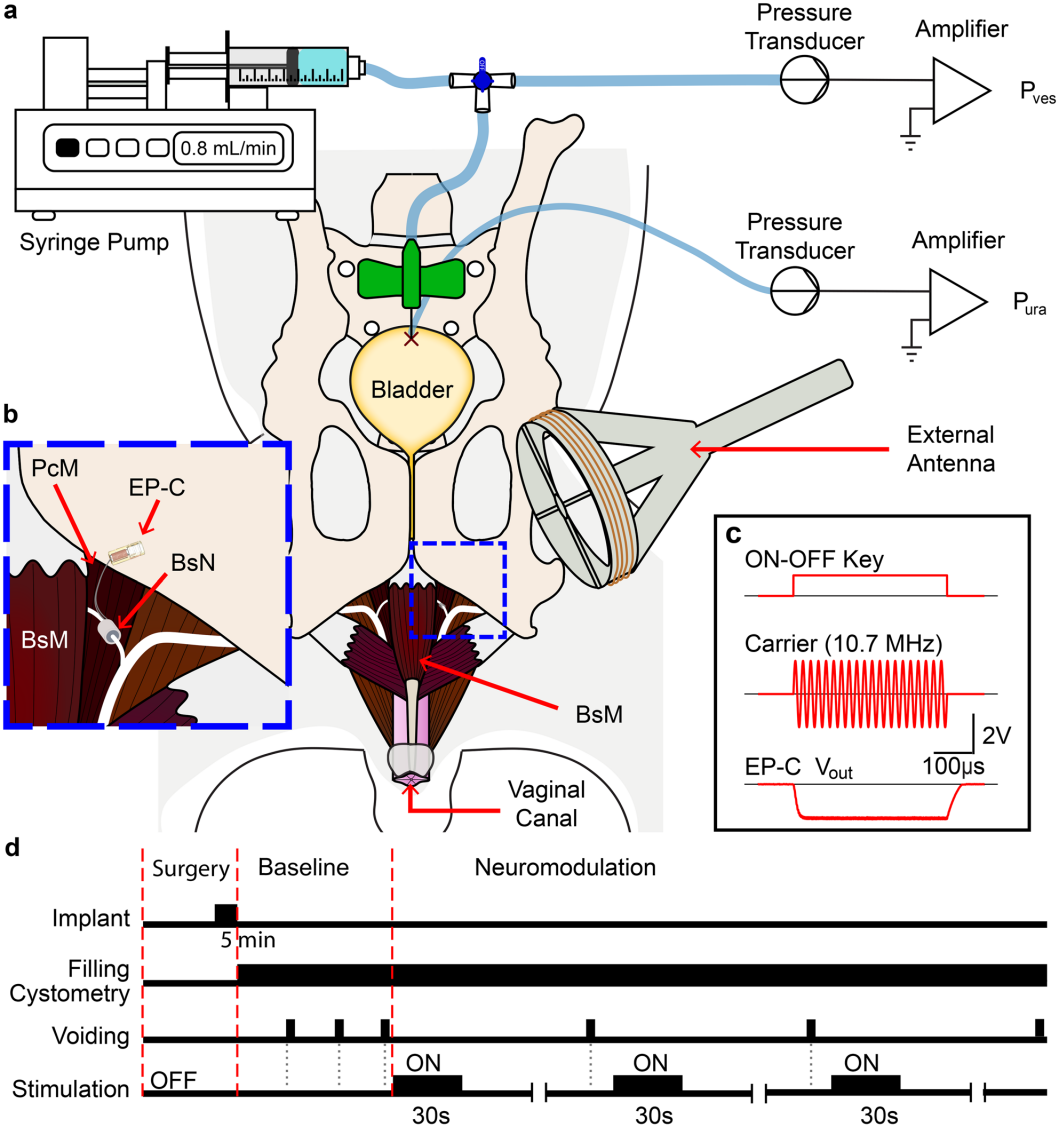
Schematic of experimental setup and wireless electrical stimulation protocol. (**a**) Cystometry setup showing suprapubic vesical (P_ves_) and urethral (P_ura_) pressures connected to a syringe pump for filling and pressure transducers. (**b**) Electroparticle-cuff nerve stimulator (EP-C) implanted on the bulbospongiosus nerve (BsN). (**c**) Electromagnetic ON–OFF keying modulation to wirelessly power the EP-C via an external antenna at a 10.7 MHz carrier resonant frequency. (**d**) Timing of electrical stimulation and cystometry studies. A baseline is recorded in triplicate and compared to EP-C 30-s stimulation in triplicate.

The morphometric analysis in this study showed that the nerve injury from multiparity is partial in these animals and that some spontaneous regeneration occurs. These observations suggested the possibility that acute electrical stimulation to these nerves may be able to increase pelvic muscle contraction. To test this hypothesis, we selected the bulbospongiosus nerve as the target of stimulation due to its superficial location compared to the pubococcygeus nerve and the well-characterized activity of this muscle during the voiding phase of the rabbit model^15^. To achieve optimal stimulation of the bulbospongiosus nerve, we used a miniature wireless stimulator connected to a commercial nerve cuff electrode (electroparticle cuff electrode; EP-cuff) that we reported recently^26^ (Fig. 8a–c).

The effect of the bulbospongiosus electrical stimulation was compared between young nulliparous and mid-age multiparous animals, and the effect determined in triplicate by cystometry (Fig. 8d). Stimulation was achieved using cathodic pulses of 92–104 µA applied directly to the nerve using the EP-Cuff (43.9 and 164.52 A/m magnetic field), either at low (2–5 Hz) or high (10–20 Hz) frequencies. Simultaneous recordings of the bladder (P_ves_) and urethra (P_ura_) pressures were obtained, and micturition volume measured.

**Figure 9:**
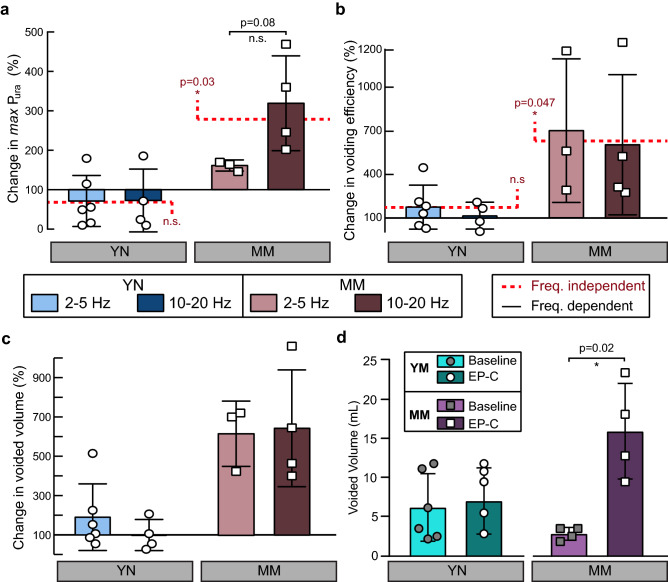
Effect of acute stimulation of the bulbospongiousus nerve in urethral pressure and urodynamics in young nulliparous (YN) and mid-age multiparous (MM) rabbits. Percent changes in (**a**) the maximum urethral pressure (max Pura), (**b**) voiding efficiency, (**c**) voided volume, and (**d**) actual voided volumes at baseline and during stimulation. All results are reported as mean ± SD dependent of frequency and the red dashed line represents the change with respect to baseline independent of stimulating frequency. Changes are not-significant (n.s.) unless otherwise noted. *p < 0.05 as per one-sample T-test for frequency independent changes (μ_o_ = 100%), and two-sample T-test for within frequencies for a single group.

Wireless electrical stimulation of the bulbospongiosus nerve did not significantly affect the urodynamics in young nulliparous animals. Conversely, we observed a threefold increase in the *max* P_ura_ in the mid-age multiparous animals resulting from the wireless stimulation at higher frequencies, which contributed to a corresponding increase in bladder volume, voided volume, and voiding efficiency. Cystometry values varied 66.0% at baseline and 44% during stimulation in the control animals, compared to 50.0% at baseline and 47.3% during stimulation in the mid-age multiparous rabbits. To analyze this data, we normalized the changes per animal as the ratio between the quantified urodynamics during wireless stimulation and baseline (Fig. 9).

This analysis showed that maximal urethral pressure (*max* P_ura_), voided volume, and voiding efficiency did not change significantly after bulbospongiosus nerve stimulation in the young nulliparous animals.

In sharp contrast, wireless stimulation with frequencies between 2–20 Hz (denoted by the red dashed line in Fig. 9) significantly increased the *max* P_ura_ (*p* = 0.04), voided volume (*p* = 0.008), and voiding efficiency (*p* = 0.047). A comparison between the 2–5 Hz and 10–20 Hz stimulation indicated that these two frequencies did not have a significant differential effect, although the *max* P_ura_ had a trend towards higher values at 10–20 Hz stimulation. The data confirmed that acute wireless electrical stimulation can recruit axons in the mid-age multiparous bulbospongiosus nerve, despite partial injury, and is effective in increasing urethral pressure, voided volume, and voiding efficiency in these animals.

## Discussion

Morphometric evaluation of the bulbospongiosus and pubococcygeus nerves in the young and mid-age multiparous animals revealed mild nerve damage caused by multiparity in young animals and confirmed the expected injury to myelinated axons in the aging multiparous rabbits. We found that axon nerve composition in the healthy pubococcygeus nerve in young nulliparous rabbits, was characterized by a larger population of unmyelinated axons compared to the bulbospongiosus nerve by almost a two-fold increase in density per 100 µm^2^. Multiparity in young rabbits showed a 10–20% reduction in axon diameter, a proportional increase in myelinated axon density, and a trend of reduced thickness of the small diameter axons in the nerves, albeit none of these changes achieved statistical significance. While nerve damage in this animal group was expected, as nerves are known to stretch beyond elastic limits during parity and delivery, the mild nature of the nerve damage is likely explained by nerve regeneration, as well as hormonal and protein adaptation during pregnancy that might be neuroprotective^27,28^.

In contrast, moderate nerve damage was observed in the aging multiparous rabbits, indicated by myelin thickening resulting from sheath decompaction, ovoid formation, recurrent looping, and Wallerian axon degeneration. Our quantitative analysis confirmed a fold increase in myelinated axon density, which covered 60% more area, and thicker myelination as shown by a significant decrease in the G-ratio. This moderate injury in the mid-age multiparous rabbits seems to indicate long-term effects of aging, including compromised repair mechanisms. While this notion is consistent with clinical reports, showing that nulliparous middle-aged women have high incidence of SUI^29,30^, the lack of mid-age nulliparous animals in this study limits this interpretation of our results.

In addition, it is well established^31–35^ that the axon diameter, myelin thickness, and internodal length play a crucial role in axon conduction velocity, and determine electrical stimulation thresholds (i.e., large diameter axons are depolarized first). During motor axon regeneration, re-growing axons are small in diameter, have an even distribution of ion channels in the membrane, and are initially unmyelinated. As remyelination starts and axons mature, ion channels clusters at the node of Ranvier, and conduction velocity increases, albeit less than pre-injury levels due to shorter internodal lengths^31^ and ectopic ion channel clustering^36,37^. Due to those changes, regenerating motor axons are expected to have higher activation thresholds and slower conduction velocities. We observed axons in the mid-age multiparous animals with large axon diameters and thin myelin, a hallmark of axonal regeneration. We also confirmed an increase in the number of small myelinated axons, some of which are expected to be regenerating axons. Therefore, histomorphometry of pelvic and perineal nerves in the mid-age multiparous animals anticipate that they will require a higher stimulation amplitude for maximal muscle contraction compared to non-injured controls. This is in agreement with our previous reports in which depolarization of these nerves in multiparous animals require twofold increased stimulating amplitude compared to normal controls^15,18,21^. Given that the bulbospongiosus nerve was most drastically affected in the aging multiparous population, we evaluated whether the acute stimulation of this nerve using a wireless implantable device could be used to activate their target muscle and to strengthen its urethral sphincter function.

As expected, baseline cystometry in mid-age multiparous animals confirmed a reduced urethral pressure and voiding efficiency, indicating partial impairment of the pelvic floor muscles. Due to the expected partial atrophy of the pelvic floor muscles^21^, electrical stimulation was done at low (2–5 Hz) and moderate (10–20 Hz) frequencies to prevent excessive stimulation or fatigue. Remarkably, acute wireless electrical stimulation of the bulbospongiosus nerve in these animals increased the maximal urethral pressure and voiding efficiency significantly, proportional to the stimulation frequency. Acute stimulation was sufficient to induce a 3-fold increase in urethral pressure and voiding volume, indicating that activation of this perineal nerve strengthens the urethral sphincter supporting continence and voiding efficiency, despite partial nerve damage. While this evidences the benefit of acute stimulation, chronic studies in fully awake animals are needed to completely understand the clinical potential of pelvic floor neuromodulation. Mechanistically, the simple interpretation of our results is that depolarization of the motor efferents directly activated the target muscle. However, further studies are needed to elucidate possible effects of the sensory afferents into the spinal cord^38^. While the acute benefit of direct nerve stimulation is compelling, understanding the mechanism and potential of this neuromodulation option for the pelvic floor will require future studies that investigate other perineal and pelvic floor nerve and muscle targets, including the puborectalis nerve and its possible effect on fecal incontinence in a chronic setting. In addition, while the observed effect on mid-age rabbits is significant, further studies are needed to determine if these beneficial effects can be confirmed in older animals, where muscle atrophy and fibrosis may complicate this approach. However, electrical stimulation has been shown to reverse muscle atrophy in the elderly^39^, which could mitigate these complications. An additional limitation to the study is the anatomical difference between the rabbit and human urogenital system. The rat model has been commonly studied to evaluate bladder and pelvic floor function^40^; in this study, the rabbit was chosen due to the larger pelvic floor muscles, and anatomical and functional characterization of the multiparity and aging effects that includes neural dysfunction in nerve conduction, muscle damage, and urethral and bladder abnormalities^22^.

Together, our results suggest that neuromodulation of the perineal and pelvic nerves that directly control individual muscles can be used for the selective treatment of SUI and other pelvic dysfunctions. However, a limitation of such an approach is the fact that this potential treatment requires the implantation of a wireless nerve stimulator. This risk is partially mitigated by current and future progress in the miniaturization of these devices. Transcutaneous and transvaginal devices have shown some efficacy in stimulating the pelvic floor in women with SUI^41^, but these treatments are not selective and less effective in elderly patients^42^. Furthermore, comparative studies between surface and implanted electrodes for nerve stimulation have demonstrated lower activation thresholds, increase selectivity, and a significant repair benefit for the implantable devices^43^. Therefore, we anticipate that patients that do not benefit from pelvic floor exercise or non-selective surface or vaginal electrical stimulation might consider a minimally invasive surgical procedure for the implantation of miniature neural stimulator in perineal and pelvic nerves. Direct nerve stimulation have the added benefit of enhancing nerve repair mechanisms, as previously reported^44^.

In summary, we characterized the partial perineal and pelvic nerve damage due to parity and aging in rabbits and demonstrated the possibility of using electrical stimulation of these nerves to improve urethral function and voiding efficiency. These findings support the possibility of using targeted pelvic floor neuromodulation as a novel approach for the management of SUI. Future studies are needed to investigate whether these effects can be confirmed in fully awake animals and if the benefit can be long-lasting.

## Materials and methods

### Animal use

A total of 27 adult chinchilla female rabbits (*Oryctolagus cuniculus*) divided in two cohorts were used. In the first cohort of 17 animals, we evaluated the extent and variability of nerve damage histologically in young (10–12 months old) multiparous (n = 7) rabbits, compared to control young nulliparous (zero pregnancies) rabbits (n = 6), and in mid-age (4-years old) multiparous (4 pregnancies; n = 4) rabbits. Multiparous rabbits began copulation at 6 months of age and mated again on the day after each delivery. They were pregnant and lactating for twenty days when pups were weaned, as it occurs in normal, natural conditions. On the day of the fourth delivery, neonate pups were euthanized to avoid lactation and to allow multiparous rabbits to set their hormonal conditions similar to nulliparous animals, as reported for serum estradiol levels^22,45^. The average litter size in multiparous animals was 6 pups (5, 7, 7, 6 for each animal per delivery, respectively). Mid-age multiparous rabbits were aged for 34 months before the experiments. We collected the bulbospongiosus and pubococcygeus nerves from the 17 animals of the first cohort. The second cohort included 10 animals which were used to evaluate the effect of neuromodulation of the bulbospongiosus nerve on voiding efficiency and urethral closure in the young nulliparous (n = 6), and mid-age multiparous (n = 4) groups.

### Nerve histomorphology

Fresh bulbospongiosus and pubococcygeus nerves of rabbits in the first cohort (n = 17) were collected and prepared for TEM as previously described^18^. Briefly, nerve samples were post-fixed in Karnovsky buffer, incubated in Zetterquist buffer, dehydrated, infiltrated with Epon-acetonitrile, and embedded in Epon at 60 °C. Transverse thin sections were obtained and imaged with JEOL 1200EX microscope (JEOL USA Inc., Peabody, Massachusetts, USA).

Quantitative analysis of TEM images was done in duplicate by two investigators blinded to the animal groups (H-R., A. and H, A.) using the freehand selection tool in Fiji:ImageJ (ImageJ version 1.52, Wayne Rasband National Institutes of Health, USA: https://imagej.nih.gov/ij)^46,47^ to outline the fiber and the surrounding myelin of each single axon. The measure tool was then used to obtain the axon fiber diameter (*D*_*axon*_), its surrounding major (*D*_*myelin*_) and minor (*d*_*myelin*_) myelin diameters, the myelin perimeter (*P*_*myelin*_), and the area of each axon. Myelination was quantified using the G-ratio, calculated as shown in Eq. (1); myelin circularity was quantified as the elliptical ratio (Φ)^48^ in Eq. (2) of the *P*_*myelin*_ divided by the calculated perimeter of an ellipse (*P*_*ellipse*_) of *D*_*myelin*_ and *d*_*myelin*_ dimensions. *P*_*ellipse*_ was calculated using the Ramanujan’s approximation^49^ in Eq. (3), where *r*_*M*_ and *r*_*m*_ are the radius of *D*_*myelin*_ and *d*_*myelin*_ respectively. All results are reported as the mean ± standard deviation. Axons that were only partially seen (i.e., part of the axon or myelin was outside of the TEM field of view) were excluded from analysis.

1 $$G = \frac{{D_{axon} }}{{D_{myelin} }}$$

2 $${\Phi } = \frac{{P_{myelin} }}{{P_{ellipse} }}$$

3 $$P_{ellipse} = \pi \left( {r_{M} + r_{m} } \right)\left( {1 + \frac{{3\frac{{\left( {r_{M} - r_{m} } \right)^{2} }}{{\left( {r_{M} + r_{m} } \right)^{2} }}}}{{10 + \sqrt {4 - 3\frac{{\left( {r_{M} - r_{m} } \right)^{2} }}{{\left( {r_{M} + r_{m} } \right)^{2} }}} }}} \right)$$

### Urodynamics

The rabbits in the second cohort were anesthetized using a 20% urethane solution (IP 1.5 g/kg) and placed in supine position. The use of urethane for this study was selected to preserve reflex functions and facilitate the measurement of the urethral pressure and cystometry. The surgical procedure was described elsewhere^50^. Briefly, a 3–4 cm incision was made to expose the bladder, and a 21Gx19mm butterfly needle was inserted at the dome and secured using a purse-string suture. This was connected to a pressure transducer (Statham Hato Rey, P23BC) and signal acquisition system (Grass 7P1B DC amplifier) to measure bladder vesical pressure (P_ves_). A balloon-catheter (1.6 mm and 1.2 mm outer and inner diameters, respectively) was introduced into the urethra and inflated using warm saline, reaching a 4.5 ± 0.70 mm diameter partially obstructing the urine flow. The balloon catheter was connected to a pressure transducer (Grass PT 300) which was connected to a signal acquisition system (Grass 7P1B DC amplifier) to record the urethral pressure (P_ura_), as previously described^22^. Urodynamic data was visualized and quantified in the PolyView recorder (ver. 2.5). All results are reported as the mean ± standard deviation.

### Cystometry

Normal saline solution (0.9% NaCl) was infused at a constant rate of 0.8 mL/min at body temperature (37 °C) into the bladder. The P_ves_ and P_ura_ baselines were simultaneously recorded in triplicate during bladder filling and voiding phases following the procedures described in previous studies^15,22,51^. Briefly, the maximum urethral pressure (*max* P_ura_) during voiding, the total voided volume defined as the volume (mL) expelled through the urogenital meatus during voiding, and voiding efficiency as the percentage of total voided volume divided by the total infused saline solution at the time of micturition were calculated. Finally, the changes in urodynamics were calculated as the percent change between evoked responses with respect to baseline.

### Nerve stimulator

A wireless electroparticle-cuff electrode with a 4-cm extension wire, as described elsewhere^26^, was used for bulbospongiosus nerve stimulation. The electroparticle-cuff was inductively powered by using the methods previously described^25,26^. Briefly, a pulse generator (Agilent 81110A) is used to control stimulating pulse width and frequencies. Its output triggers a waveform generator (Agilent 33250A) with an ON–OFF keying modulation (OOK) that generates the carrier signal at a 10.7 MHz resonant frequency to which the electroparticle-cuff is tuned. Its output is amplified (T&C Power Conversion, Inc. AG series Amplified) and the signal is carried to the electroparticle-cuff by an external antenna designed to provide RF magnetic fields ranging from 16.87 to 27.5 A/m^26^. The output from the electroparticle-cuff is a voltage-controlled (V_out_) monophasic pulse delivered using a bipolar configuration by two platinum-iridium (PrIr) electrodes in the commercial nerve cuff attached to the electroparticle-cuff.

### Device implantation

After isolation of the bulbospongiosus muscle, its motor nerve is located lateral to the clitoral nerve. A 5 mm segment of the bulbospongiosus nerve was carefully isolated from the underlying tissues. We opened the cuff of the electroparticle-cuff by holding the cuff tabs and gently pulled the nerve upwards allowing it to slide into the cuff tunnel and released the tabs to secure the implant position, and the extension wire placed subcutaneously.

### Neuromodulation

All 10 rabbits in the second cohort underwent electrical stimulation of the bulbospongiosus nerve for 30 s at a low (2–5 Hz) and moderate (10–20 Hz) frequencies. These frequencies were selected to prevent overstimulation of the perineal muscles, as the aging multiparous animals are known to have some muscle atrophy based on histological and functional studies^19–21^.

The external antenna was placed 4-cm from the electroparticle-cuff and provided monophasic 400 µs wide pulses through a magnetic field that varied between 43.86 and 164.52 A/m, to provide a 20–60% power amplification of the carrier signal^26^. After every stimulation burst, the animals were given a stimulation-free window until a voiding was observed. The P_ves_ and P_ura_ were continuously recorded. The evoked response in P_ura_ was calculated as a percentage of the baseline for comparison (Q P_ura_). Animals were euthanized at the end of the experiments using an intraperitoneal (IP) overdose of urethane (2.5 g/kg).

### Data and statistical analysis

Sample sizes were calculated using data from our previous report on the percentage of young nulliparous and mid-age multiparous with myelination abnormalities in the bulbospongiosus and pubococcygeus nerves based on a qualitative analysis (15 ± 4% and 7 ± 4% in young multiparous vs. 41 ± 4% and 32 ± 4% in mid-age multiparous; respectively)^18^. For an error probability α = 0.05 and desired 0.80 power, the estimated sample size was equal to four (n = 4). The sample size provided an accurate power prediction since analysis performed in RStudio v1.3.1056 (R v3.6.1) using the nls2 (v 0.2)^52^, ggplot2 (v3.3.2)^53^, and plyr (v1.8.4)^54^ libraries, showed an ANOVA effect size = 1.2, and 0.91 and 0.87 power for the bulbospongiosus and pubococcygeus, respectively. Similarly, data on void duration in this study for α = 0.05 and 0.80 power, the sample size of n = 4 was accurate. All data was verified for normality and homogeneity using Shapiro–Wilk’s normality test, and Levene’s test of homogeneity at α = 0.05. Log-transformation was applied to non-normally distributed data and verified again for normality. Multivariate analysis of variance (MANOVA) was used to test statistical significances of G-ratio and Φ among groups followed by Tukey’s post-hoc test for multiple comparisons.

Equation (4) was used as the model for non-linear regressions, where the Y-axis = ${\text{G-ratio}}_{group}$ and the X-axis = *D*_*axon*_, *group* = [YN = young nulliparous, YM = young multiparous, MM = mid-age multiparous] is the experimental group, ${\text{G-ratio}}_{plateau}^{group}$ is the constant value the non-linear function reaches as *D*_*axon*_ increases in size, ${\text{G-ratio}}_{origin}^{group}$ is the G-ratio value at the origin, and α is the decay rate of the exponential function. The initial parameter search (R function: nls2) was done using the grid-search (brute-force) algorithm^52^. Parameter fine-tuning (R function: nls) was performed using non-linear least squares via the port algorithm^55^ with no bounds supplied.

4 $${\text{G-ratio}}_{group}\left({D}_{axon}\right)={\text{G-ratio}}_{plateau}^{group}+\left({\text{G-ratio}}_{origin}^{group}-{\text{G-ratio}}_{plateau}^{group}\right){e}^{-\propto {\bullet D}_{axon}}$$

The non-linear regression coefficients calculated for the control young nulliparous group were used to create a vector with *D*_*axon*_ = [0.1, 0.2, …, 16] μm. Another vector was created for the young multiparous and mid-age multiparous groups using their calculated coefficients and compared to the control group using the correlation coefficient (ρ) between vectors.

For the second cohort, the average for all urodynamic metrics between repeated measurements was calculated for each animal. Then, the mean and standard deviation was calculated for the sample, and the coefficient of variation was computed as the ratio between the standard deviation and the mean multiplied by 100 to determine the variation percentage for all metrics. We normalized the data as a percent change per animal with respect to baseline. We assessed the change evoked by the electrical stimulation per group according to stimulation frequency using one-sample, right-tailed T-test with a 5% significance level (*p* = 0.05) and a population mean (μ_o_) as 100%. This test informed whether the electrical stimulation value was different compared to baseline. A T-test was used then to determine the change between lower (2–5 Hz) and the higher (10–20 Hz) frequencies within group. All results are reported as mean ± SD, and p values < 0.05 were considered significant.

### Ethics appraoval

All animal experiments were performed according to the Institutional Guidelines for Treatment of Animals in Research and approved by the Animal Care and Use Committee of the Instituto de Investigaciones Biomédicas-UNAM (CICUAL 260). This work adheres to the Animal Research: Reporting of in Vivo Experiments (ARRIVE) guidelines for reproducible science and transparent reporting.

## Supplementary Information

Supplementary Information.

## Data Availability

Raw data and other pertinent information are available upon request.
