# Supplementary material for: Targeted neuromodulation of pelvic floor nerves in aging and multiparous rabbits improves continence
Source: Sci Rep. 2021 May 19;11:10615. doi: 10.1038/s41598-021-90088-8 (PMC8136474; doi:10.1038/s41598-021-90088-8)
Supplement: Supplementary file 1 — Supplementary Information. [file 41598_2021_90088_MOESM1_ESM.docx]

Supplementary information

**Targeted Neuromodulation of Pelvic Floor Nerves in Aging and Multiparous Rabbits Improves Continence**

Ana G. Hernandez-Reynoso ^1, 2, 3^, Dora L. Corona-Quintanilla^4^, Kenia López-García^5^, Ana A. Horbovetz^1^, Francisco Castelán ^4, 5^, Philippe Zimmern ^6^, Margarita Martínez-Gómez ^4, 5, #^, Mario I. Romero-Ortega ^2, 3, 7, #, *^

^1^ Department of Bioengineering, University of Texas at Dallas, Richardson, TX, 75080, USA

^2^ Department of Surgery, University of Texas Southwestern Medical Center, Dallas, 75390, USA

^3^ Department of Biomedical Engineering and Biomedical Sciences, University of Houston, TX, 77204, USA

^4^ Centro Tlaxcala de Biología de la Conducta, Universidad Autónoma de Tlaxcala, Tlaxcala, Tlaxcala, México

^5^ Departamento de Biología Celular y Fisiología, Unidad Foránea Tlaxcala, Instituto de Investigaciones Biomédicas, Universidad Autónoma de México, Tlaxcala, Tlaxcala, México

^6^ Department of Urology, University of Texas Southwestern Medical Center, Dallas, 75390, USA

^7^ Department of Health Care Sciences, University of Texas Southwestern Medical Center, Dallas, 75390, USA.

# = Equal senior investigator contribution

* = Correspondence to: Mario I. Romero-Ortega [miromer2@central.uh.edu](mailto:miromer2@central.uh.edu)

**Figure 1Supplementary**

**
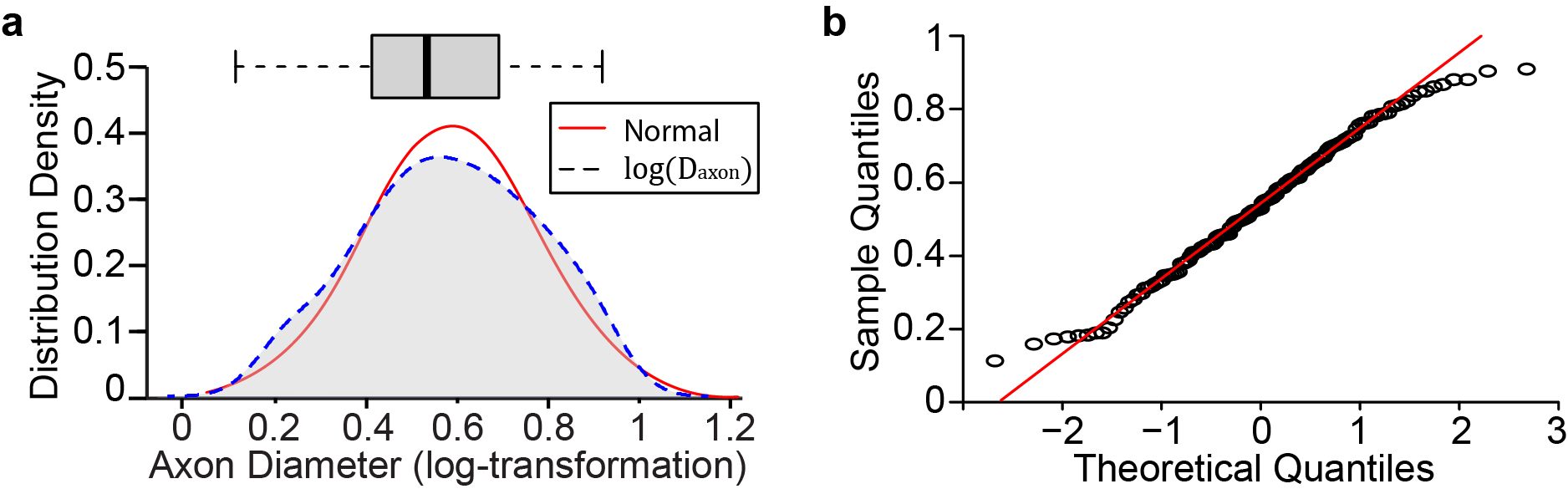
**

Distribution of log-transformed axon diameter (*D_axon_*) data (ImageJ version 1.52, Wayne Rasband National Institutes of Health, USA: <https://imagej.nih.gov/ij>) for bulbospongiosus nerve (Bsn) in middle age multiparous animals. (a) Distribution density of data overlapped with a normal distribution for comparison. The boxplot on top helps to determine normality distribution of data. (b) Quantile-quantile plot of theoretical and sample data for the log-transformed data comparing to a normal distribution. Results show that the deviations from normality are not significant, and ANOVA is feasible.

**Figure 2 Supplementary**


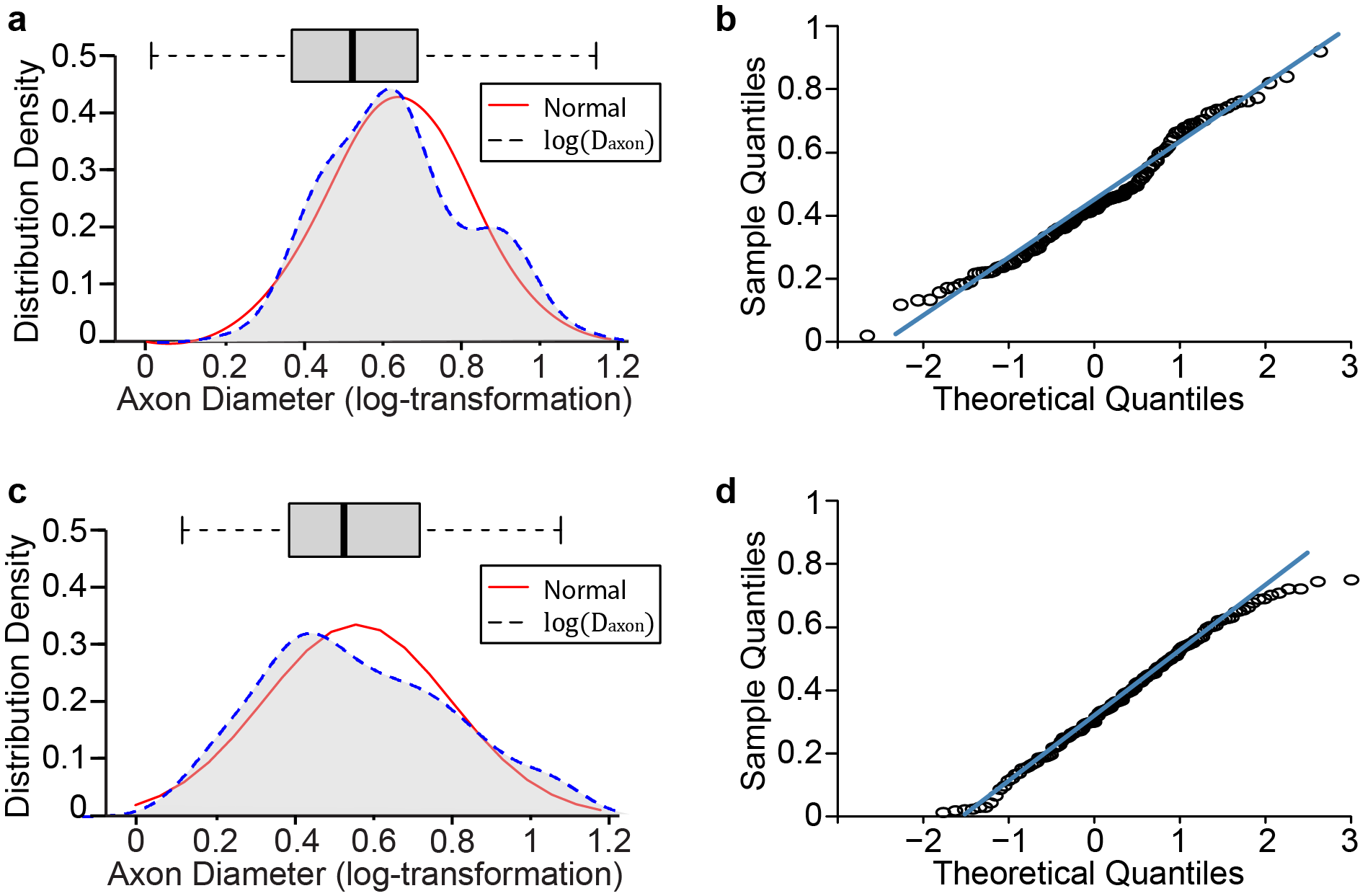


Distribution of log-transformed axon diameter (*D_axon_*) data (ImageJ version 1.52, Wayne Rasband National Institutes of Health, USA: <https://imagej.nih.gov/ij>) for pubococcygeus nerve (Bsn) in young multiparous (top) and middle age multiparous (bottom) animals. (a & c) Distribution density of data overlapped with a normal distribution for comparison. The boxplot on top helps to determine normality distribution of data. (b & d) Quantile-quantile plot of theoretical and sample data for the log-transformed data comparing to a normal distribution. Results show that the deviations from normality are not significant, and ANOVA is feasible.

**Table 1 Supplementary** Resulting coefficients of non-linear regression of the G-ratio and *D_axon_* of the bulbospongiosus nerve for all three groups: young nulliparous (YN), young multiparous (YM), and mid-age multiparous (MM).

| **Condition** | $\text{G-ratio}_{\boldsymbol{plateau}}^{\boldsymbol{group}}$ | $\text{G-ratio}_{\boldsymbol{origin}}^{\boldsymbol{group}}$ | **α** | **RSS** |
| --- | --- | --- | --- | --- |
| YN | 0.68 | -11.53 | 3.16 | 0.66 |
| YM | 0.70 | -0.02 | 1.26 | 1.18 |
| MM | 0.61 | 0.17 | 0.73 | 1.104 |

**Table 2 Supplementary** Resulting coefficients of non-linear regression of the G-ratio and *D_axon_* of the pubococcygeus nerve for all three rabbit groups: young nulliparous (YN), young multiparous (YM), and mid-age multiparous (MM).

| **Condition** | $\text{G-ratio}_{\boldsymbol{plateau}}^{\boldsymbol{group}}$ | $\text{G-ratio}_{\boldsymbol{origin}}^{\boldsymbol{group}}$ | **α** | **RSS** |
| --- | --- | --- | --- | --- |
| YN | 0.69 | -0.54 | 1.85 | 0.88 |
| YM | 0.70 | -1.79e-3 | 5.68 | 0.83 |
| MM | 0.74 | 0.53 | 0.10 | 1.84 |

**Table 3 Supplementary.** Quantification of urodynamic variables in baseline and stimulation. Analysis of urodynamics during stimulation was performed for overall stimulation (2-20 Hz) to determine its effect, and was then stratified do compare lower (2-5 Hz) vs. higher frequencies (10-20 Hz).

|  |  | **Baseline** | **2-20 Hz** | **2-5 Hz** | **10-20 Hz** |
| --- | --- | --- | --- | --- | --- |
| ***max* P_ura_ (cmH_2_O)** | YN | 9.64±8.83 | 2.89±1.44 | 3.06±1.58 | 2.48±1.30 |
|  | MM | 3.00±1.68 | 9.94±10.07 | 3.54±1.21 | 11.07±9.78 |
|  |  |  |  |  |  |
| **Volume at micturition** | YN | 14.06±8.91 | 12.24±4.29 | 12.18±4.02 | 11.50±6.76 |
| **(mL)** | MM | 25.25±14.08 | 28.4±8.59 | 28.67±2.52 | 29.92±10.14 |
| **Voided volume (mL)** | YN | 6.39±4.33 | 8.22±3.74 | 8.67±3.94 | 5.88±4.97 |
|  | MM | 2.42±0.93 | 15.15±6.35 | 15.83±2.02 | 15.29±8.43 |
|  |  |  |  |  |  |
| **Voiding efficiency (%)** | YN | 46.25±19.16 | 56.71±25.91 | 57.68±25.65 | 47.87±30.07 |
|  | MM | 10.75±5.35 | 52.81±8.37 | 55.37±3.61 | 50.05±15.82 |
